# Supplementary material for: Selection for feed efficiency elicits different postprandial plasma metabolite profiles in response to poor hygiene of housing conditions in growing pigs
Source: PLoS One. 2021 Mar 29;16(3):e0246216. doi: 10.1371/journal.pone.0246216 (PMC8006997; doi:10.1371/journal.pone.0246216)
Supplement: Attachment — Submitted filename: Response to Reviewers_def.docx [file pone.0246216.s001.docx]

Dear Editor,

We greatly thank the reviewer for the critical and helpful comments on our manuscript that significantly contributed to this new version. As you will see, we carefully addressed each of the reviewer’s comments and we hope that all changes will meet your expectations.

Our answers are listed below and a version of the revised manuscript with all the modifications (i.e., corrections and additional information inserted) highlighted in red is provided.

Yours sincerely,

Nathalie Le Floc’h

**#Journal Requirements:**

When submitting your revision, we need you to address these additional requirements.

1) Please ensure that your manuscript meets PLOS ONE's style requirements, including those for file naming. The PLOS ONE style templates can be found at <https://journals.plos.org/plosone/s/file?id=wjVg/PLOSOne_formatting_sample_main_body.pdf> and <https://journals.plos.org/plosone/s/file?id=ba62/PLOSOne_formatting_sample_title_authors_affiliations.pdf>

*Authors:* *The entire manuscript was revised and authors' affiliations were corrected* *to meet PLOS ONE’s style requirements (lines 6-12). Information regarding funding and author contributions were also included (lines 438-454).*

2) In your Methods, please provide full details of animal care and housing.

*Authors:* *Additional information in the Material and Methods section was included according to the journal's requirements (lines 76-78 and 97-98).*

3) Thank you for including your ethics statement:  "The experiment was conducted at INRAE UE3P (Saint-Gilles, France) in accordance with the ethical standards of the European Community (Directive 2010/63/EU), and was approved by the Regional ethical committee (CREEA number 07). The experiment approval number is APAFIS#494-2015082717314985.".

Please amend your current ethics statement to include the full name of the ethics committee that approved your specific study. For additional information about PLOS ONE submissions requirements for ethics oversight of animal work, please refer to <http://journals.plos.org/plosone/s/submission-guidelines#loc-animal-research>

Once you have amended this/these statement(s) in the Methods section of the manuscript, please add the same text to the “Ethics Statement” field of the submission form (via “Edit Submission”).

*Authors:* *The information regarding the experimental approval number was indicated according to the journal's requirements (lines 76-78).*

4) We note that the grant information you provided in the ‘Funding Information’ and ‘Financial Disclosure’ sections do not match. When you resubmit, please ensure that you provide the correct grant numbers for the awards you received for your study in the ‘Funding Information’ section.

*Authors: Information regarding “Funding” was indicated according to the journal's requirements (line 438-443).*

5) We note that you have included the phrase “data not shown” in your manuscript. Unfortunately, this does not meet our data sharing requirements. PLOS does not permit references to inaccessible data. We require that authors provide all relevant data within the paper, Supporting Information files, or in an acceptable, public repository. Please add a citation to support this phrase or upload the data that corresponds with these findings to a stable repository (such as Figshare or Dryad) and provide and URLs, DOIs, or accession numbers that may be used to access these data. Or, if the data are not a core part of the research being presented in your study, we ask that you remove the phrase that refers to these data.

*Authors: This sentence was rephrased and values for ADG have been added in the text (lines 199 and 200).*

__________________________________________________________________

Reviewers' comments:

**Reviewer #1:**

Overall an interesting study with interesting results. There are however some major and minor changes to make before it can be published. Some explanations are missing and some inconsistencies need to be solved first.

**Major remarks:**

1. Be consistent in terminology, in the manuscript more different terms are used to indicate the same treatment. This is confusing and should be more consistent. For example; In the title you write ‘poor hygiene of housing conditions’, L19 ‘poor environmental conditions’, L31/32 ‘poor hygiene conditions’, 111 ‘health status’, L117 ’Hygiene challenge’ etc.

*Authors:* *We totally agree with the reviewer’s suggestion. To avoid confusion, the entire manuscript was revised and only one term was used (“poor hygiene of housing conditions”).*

2. In the conclusion is stated (L415/416): …’which is in line with the greater impact of the challenge on protein deposition’. I don’t think this can be stated as there is no significant effect on ADG of the pigs in this experiment. So, this should be deleted.

*Authors:* *We totally agree with this comment. The sentence was rephrased since it referred to the previous experiment and not to that on (lines 428-430). As already mentioned in the material and methods section, the present experiment was performed on a subset of pigs from a larger study (Chatelet et al. 2018) showing a clear interaction between genetic lines and hygiene of housing. In the present experiment, despite a numeric difference, it was not possible to confirm such a significant effect on ADG due to the low number of pigs (6 instead of 40 per experimental group; lines 339-341).*

3. In the manuscript several times the word ‘hyper-activation’ is used. I think ‘hyper’ is suggesting the wrong thing, such as over-activation. Activation without hyper will do.

*Authors: We changed to “activation” throughout the entire manuscript according to reviewer’s suggestion.*

4. L59 this sentence suggests that this study is already done and known. Make clear why /what is new in your study.

*Authors:* *We agree with this comment. To avoid misunderstandings and to clarify our study, the entire paragraph was reformulated according to the reviewer’s suggestion (lines 58-60; 63-65).*

*The current study was conducted on a subset of 24 Large-White pigs from a previous experiment already published (lines 80 and 81; Chatelet et al., 2018 Animal 12:350–358; n=160 pigs).*

*As previously reported by Chatelet et al. (2018), LRFI pigs has better ability compared with HRFI growing pigs to cope with an immune challenge caused by poor hygiene of housing conditions. In the current study, we hypothesized that this difference in coping ability between RFI pigs may involve changes in their metabolism after a 6-week exposure to contrasted hygiene of housing conditions (lines 60-62). For this, at week 6, the 24 selected pigs (n = 6 per experimental group) were fitted with an intravenous ear catheter. The evaluation was performed through a serial evaluation (0, 10, 20, 30, 40, 50, 60, 75, 90, 105, 120, 150, 180, 210 and 240 min relative to the meal delivery) of insulin and nutrients postprandial concentrations in order to compare metabolic modifications induced by poor hygiene of housing conditions in LRFI and HRFI pigs (lines 128-132 and 309-315).*

**Minor remarks:**

L25 (W3), and

*Authors:* *Corrected according to the reviewer’s suggestion (line 22).*

L43 delete ‘the’ at ‘with the poor hygiene’…

*Authors: Corrected according to the reviewer’s suggestion (line 40).*

L47 you write; ‘growth and maintenance’ but shouldn’t it we maintenance only? The next sentence explains it by heat production, physical activity and metabolism, so I understand only directly on maintenance and indirectly on growth.

*Authors: The initial sentence was correct. Estimation of feed intake is based on both growth and maintenance. The sentence was slightly modified for more clarity (lines 44-45).*

L53 ‘like’ should be ‘such as’

*Authors: Corrected according to the reviewer’s suggestion (line 51).*

L56 results

*Authors: Corrected according to the reviewer’s suggestion (line 53).*

L56/57 delete ‘to support the immune responses’

*Authors: Deleted according to the reviewer’s suggestion.*

L57 reduces

*Authors: Corrected according to the reviewer’s suggestion (line 54).*

L63 add ‘,and nitrogen- and energy-related’

*Authors: The sentence was reformulated according to the reviewer’s suggestion (lines 64-65). “Nitrogen related metabolites” has been replaced by “urea and amino acids”.*

L64 metabolites, as it

*Authors: Corrected according to the reviewer’s suggestion (line 65).*

L79 body weight (BW)

*Authors: Corrected according to the reviewer’s suggestion (line 82).*

L84 95 kg BW

*Authors: Corrected according to the reviewer’s suggestion (line 88).*

L91-93 How were cleaning, and disinfection done, and precautions taken?

*Authors: This information was included according to the reviewer’s comments (lines 97-98). In good hygiene conditions, there was no mixing with non-experimental pigs and the staff put clean boots, clothes and gloves before entering the room.* *Strict biosecurity precautions were done before and daily throughout the experimental period.*

L98 very brief information of the diet, make it more detailed. L98 complete soya beans or soy bean meal? What time of bran? Ricebran?

*Authors: Additional information was provided according to the reviewer’s suggestion (Table 1; line 113).*

L99 meet or exceed is quite vague. Perhaps it is better to at a table with the exact diet.

*Authors:* *The table with ingredients and chemical composition information of the diet was added according to the reviewer’s suggestion (Table 1; lines 103 and 113).*

L101 L-methionine or DL-methionine?

*Authors:* *DL-methionine. This information was included according to the reviewer’s request (Table 1).*

L113-L116 very long sentence

*Authors: The sentence was reformulated according to the reviewer’s suggestion (lines 123-124).*

L120 15% meal size? On average for all pigs? Maintenance energy for poor might be different than for good conditions.

*Authors: We fully agree with the reviewer’s comment that energy requirement for both maintenance and growth may be influenced by hygiene conditions and this did not escape our attention. However, in the current study, the standard feed was formulated to meet the nutritional requirement of growing pigs regardless of hygiene conditions.*

*We would like to highlight that in our experimental conditions, postprandial evaluation of insulin, energy and AA were performed using a very specific, complex and well-thought protocol in which after being overnight fasted, pigs received and ingested the same amount (300 g) of the same feed. That is, pigs were in a similar nutritional status (overnight fasted pigs), received and ingested the same amount of feed (300 g) in a very specific time-interval (10 minutes; line 131). Thus, differences in plasma profiles were associated to differences in both digestion and postprandial metabolism induced by the experimental factors,* *namely the genetic line, the hygiene of housing condition, and their interaction (lines 310-314). Feeding the pigs with different energy or amino acid content according to the experimental factors (genetic line and hygiene) would have added a third factor of variation that make the interpretation of our results even more difficult.*

L121 ADFI

*Authors:* *Corrected according to the reviewer’s suggestion (line 129).*

L114 There is written ‘assay sensitivity’ but it is not clear to what assay it is referring to.

*Authors:* *We changed to “assay sensitivity for insulin” according to reviewer’s suggestion (line 153).*

L189-190 No effect on ADG? Explain in discussion what can be the reason. A difference in ADG is expected right? I would expect a difference in ADG especially in the starter phase (25 kg-60kg). 6 weeks is also long enough to see a clear effect normally. What about an effect on ADG for LRFI of HRFI pigs? This is not presented but should at least be written in the results.

*Authors: The values for ADG measured in poor and good hygiene are now indicated in the text (lines 199-200). The numeric difference does not reach the level of significance and the main explanation is the low number of pigs. Regarding the difference between RFI pigs, as our main objective was not to compare the RFI lines but their response to the challenge (lines 69-72, 185-186 and 300-303), we preferred to keep the original sentence and do not add the ADG comparison between RFI lines.*

L194, L196 Be consistent with p-values P<0.05 or p = 0.04, both significant why showing them in a different way?

*Authors: Thanks for the comment. However, on line 204, we are discussing the results of two different variables (that is, plasma haptoglobin concentrations and blood neutrophil granulocyte counts). In contrast, on line 206, the p-value=0.04 refers to a single comparison* *of a single variable (good vs poor hygiene conditions for total IgG plasma concentrations).*

L197 write the letters of the figure in capitals, as is done in the figures.

*Authors: Corrected according to the reviewer’s suggestion (lines 204-206; 239-240; and 294-295).*

Table 1. and other tables. Is it an option to show non-significant values as n.s.?

*Authors: Thanks for the suggestion. However, we prefer to keep the values in the table to highlight the P-value number.*

L295 nitrogen-related metabolites comes out of the blue for me, please explain also in introduction.

*Authors: Corrected for urea and amino acids according to the reviewer’s comment (line 309).*

L297 Briefly, after being fasted overnight, on the day of serial blood sampling, all pigs….

*Authors: Corrected according to the reviewer’s suggestion (line 311).*

L303 how do you know this is inflammation? Was fever measured?

*Authors:* *Thanks for the comment. The findings of greater plasma haptoglobin concentrations and blood neutrophil granulocyte counts in poor than in good hygiene of housing conditions at W3 indicate that the model successfully induced an inflammation and activated immune responses. Indeed, haptoglobin a major acute phase protein in pig, and blood neutrophil granulocyte counts are both relevant indicators of inflammation in pigs (Eckersall PD, Bell R Vet J. 2010; 185: 23-27 and Odink et al., 1990; J Anim Sci. 68:163-170).*

*Therefore, we prefer to keep the term inflammation, but additional information was included* *according to the reviewer’s comment (lines 321-323).*

L303 ‘health’ this term is difficult to use here. With an activated immune system you can be perfectly healthy….

*Authors: Corrected according to the reviewer’s suggestion (line 341).*

L304 ‘decreases pig performance’, this was not the case in the current study. No effect on ADG was found for poor or good conditions.

*Authors: The sentence was deleted according to the reviewer’s comment.*

L307 ‘inflammation’… I would suggest to use immune activation and not inflammation. No body temperature was measured.

*Authors: Thanks for the comment but we prefer to keep the original sentence.*

*As we previously mentioned:*

*The findings of greater plasma haptoglobin concentrations and blood neutrophil granulocyte counts in poor than in good hygiene of housing conditions at W3 indicate that the model successfully induced an inflammation and activated immune responses. Indeed, haptoglobin a major acute phase protein in pig, and blood neutrophil granulocyte counts are both relevant indicators of inflammation in pigs (Eckersall PD, Bell R Vet J. 2010; 185: 23-27 and Odink et al., 1990; J Anim Sci. 68:163-170).*

L308 overstimulated should be stimulated

*Authors: Corrected according to the reviewer’s suggestion (line 324).*

L316 ‘showing the fast return to glucose homeostasis’ or a too low challenging effect…?

*Authors:* *Thanks for the comment. Fast return to glucose homeostasis was observed by Merlot et al., 2016 J Anim Sci 94: 563-573 in study whose pigs were challenge with complete Freund’s adjuvant. In the current study, greater values of neutrophil granulocyte count and haptoglobin concentrations at W3 and greater IgG plasma concentrations at W6 in poor than in good hygiene conditions indicate that the model of sanitary challenge successfully induced pigs immune responses.*

*Still, the same model of poor hygiene of housing conditions was also used in other studies such as Le Floc’h et al., 2014 Animal. 8: 1632-1642 and Montagne et al., 2012 J Anim Sci* *90:2556–2569, in which an increase in immune response indicators (Le Floc’h et al., 2014) and a greater incidence of digestive disorders (Montagne et a., 2012) was observed in poor than in good hygiene of housing conditions.*

*Therefore, we think that the challenge effect was moderate but induced long lasting and irreversible effects (no recovery growth rate and pneumonia lesions at slaughter) in our experimental conditions. However, metabolic effect (glucose) and blood indicators of immune activation seem to exhibit more transient changes at the blood level.*

L319 lower AA retention as muscle protein, if so an effect on ADG should be present and this was not the case…

*Authors: Thanks for the comment. We interpret that the greater concentrations of Ala, His, Met, Thr and BCAA for pigs in good than in poor hygiene conditions may be due to a lower AA muscle protein in immune challenged pigs caused by an increase in protein breakdown and/or decrease in protein synthesis, as reported by McGilvray et al., 2019 Animals. 9: 323-339.*

*As previously mentioned (please see #2 of major remarks):*

*The present experiment was performed on a subset of pigs from a larger study (Chatelet et al. 2018) showing a clear interaction between genetic lines and hygiene of housing. In the present experiment, despite a numeric difference, it was not possible to confirm such a significant effect on ADG due to the low number of pigs (6 instead of 40 per experimental group; lines 337-338).*

L410 metabolites

*Authors: Corrected according to the reviewer’s suggestion (Line 428).*

L414 ‘and growth’ should be deleted (no effect on ADG was found…)

*Authors: Deleted according to the reviewer’s suggestion.*

L416 Impact of challenge on protein deposition? This cannot be stated as there is no data supporting a difference. Or you have to show data with difference for protein deposition of difference in ADG.

*Authors:* *We agree with the reviewer’s comment. The sentence was rephrased since it referred to the previous experiment and not to that on (lines 434-435).*

__________________________________________________________________

6. PLOS authors have the option to publish the peer review history of their article (what does this mean?). If published, this will include your full peer review and any attached files. If you choose “no”, your identity will remain anonymous but your review may still be made public.

*Authors: We agree.*
